# Thioesterase YbgC affects motility by modulating c-di-GMP levels in *Shewanella oneidensis*

**DOI:** 10.1038/s41598-017-04285-5

**Published:** 2017-06-21

**Authors:** Tong Gao, Qiu Meng, Haichun Gao

**Affiliations:** Institute of Microbiology and College of Life Sciences, Zhejiang University, Hangzhou, Zhejiang 310058 China

## Abstract

Because of ubiquity of thioesters, thioesterases play a critical role in metabolism, membrane biosynthesis, signal transduction, and gene regulation. In many bacteria, YbgC is such an enzyme, whose coding gene mostly resides in the *tol*-*pal* cluster. Although all other proteins encoded in the *tol*-*pal* cluster are clearly involved in maintaining cell envelope integrity and cell division, little is known about the physiological role of YbgC. In this study, we identify in *Shewanella oneidensis*, a γ-proteobacterium used as a research model for environmental microbes, YbgC as a motility regulator. The loss of YbgC results in enhanced motility, which is likely due to the increased rotation rate of the flagellum. The regulatory function of YbgC requires its thioesterase activity but could not be replaced by YbgC homologues of other bacteria. We further show that the regulation of YbgC is mediated by the second message c-di-GMP.

## Introduction

Gram-negative bacteria are characterized by the presence of an inner membrane (IM) and a distinct outer membrane (OM), separated by a semi-aqueous compartment termed the periplasm where the peptidoglycan (PG) layer resides^1^. These structures all together constitute the cell envelope, which is essential for survival and proliferation in environments. Integrity and function of the cell envelope in part relies on membrane-associated protein complexes that transport substrate and energy between the environment and the cytoplasm^2–4^. One such system is the Tol-Pal system; its loss causes a variety of phenotypes tied to cell envelope integrity and cell division^4–8^. Moreover, the systems are essential for the polar localization of some proteins, including chemoreceptors in *Escherichia coli* and polar localization factor TipN in *Caulobacter crescentus* ^4, 9^.

**Figure 1:**
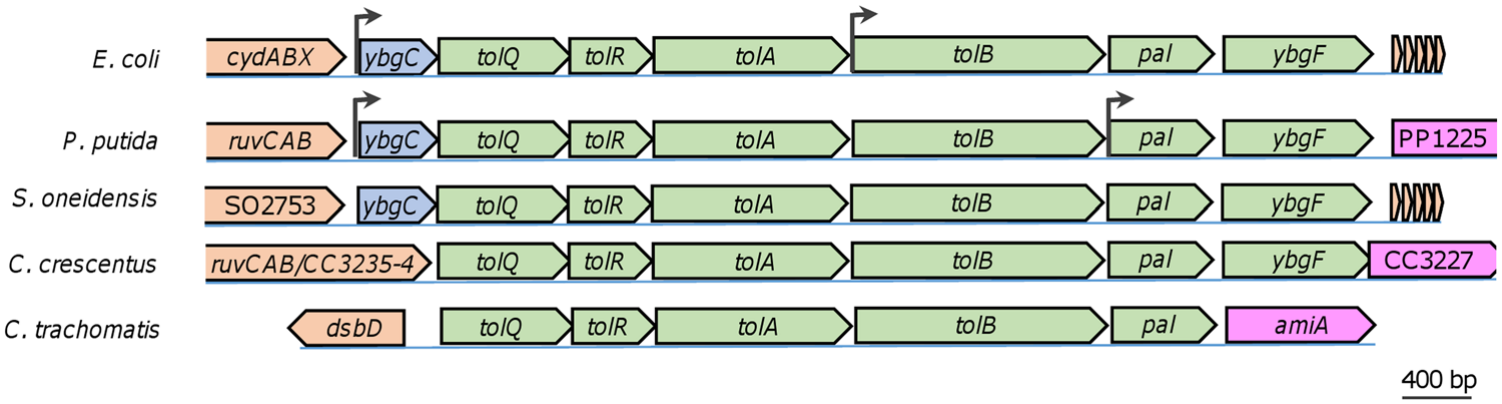
Organization of the *tol-pal* cluster in representative bacteria. Genes flanking the *tol*-*pal* cluster vary. Operon structures for the *tol-pal* cluster have been determined only in *E. coli* and *P. putida*, with promoters shown by arrows. In some bacteria, *ybgC* is missing in the *tol*-*pal* cluster and in extremely rare cases, both *ybgC* and *ybgF* are missing. Genes are drawn to scale. BLASTp E-values of *S. oneidensis* counterparts to *E. coli* YbgC, TolQ, TolR, TolA, TolB, Pal, and YbgF, are 5e-40, 7e-98, 8e-16, 2e-10, 9e-133, 8e-56, and 1e-22, respectively.

The Tol-Pal system consists of six established members, IM proteins TolA, TolQ, and TolR, periplasmic proteins TolB and YbgF, and OM protein Pal^10–12^. However, the *tol-pal* gene cluster commonly comprises seven open reading frames, *ybgC*-*tolQ*-*tolR*-*tolA*-*tolB*-*pal*-*ybgF*, although exceptions (either lacking *ybgC* or both *ybgC* and *ybgF*) are found (Fig. 1)^13^. To date, there is no evidence to support a role of YbgC in the Tol-Pal system. Operon organizations for these genes differ depending on species, for examples, two operons, *ybgC-tolQ-tolR-tolA/tolB*-*pal*-*ybgF* and *ybgC-tolQ-tolR-tolA*-*tolB/pal*-*ybgF* in *E. coli* and *Pseudomonas putida*, respectively^14, 15^. YbgC proteins, found only in bacteria, belong to the hot-dog thioesterase superfamily^16^. Consistently, esterase/thioesterase activity (acyl-CoA hydrolase) for YbgC proteins in *E. coli*, *Haemophilus influenzae*, and *Helicobacter pylori* has been demonstrated; as a consequence, it has been proposed that YbgC proteins might be involved in the biosynthesis of species-specific phospholipids^17–19^.

Bacteria have evolved complex mechanisms to control transition between the motile planktonic and sedentary biofilm-associated forms of life in response to both extra- and intra-cellular cues^20^. A key player in the decision is second messenger bis-(3′-5′)-cyclic dimeric guanosine monophosphate (c-di-GMP), which inhibits flagellar assembly and/or movement while enhancing biosynthesis of extracellular polymeric substance (EPS) required for biofilm formation^21^. A link between thioesterase and c-di-GMP has been established via diffusible signal factor (DSF) in certain bacteria^22, 23^.

*Shewanella oneidensis*, a Gram-negative γ-proteobacterium renowned for its respiratory versatility and enormous potential in bioremediation and microbial fuel cells, is now considered a research model organism in bacterial physiology^24, 25^. The bacterium is highly motile by virtue of its single polar flagellum, whose assembly has been studied extensively in recent years^26–32^. In our previous investigation into spatial and numerical control of flagellar biosynthesis, we revealed many novel features of FlhF, a protein proposed to be a determinant of polar flagellar assembly in polarly flagellated bacteria^32^. However, mechanisms underlying polar localization of FlhF and the flagellum, remain unknown.

In our continued efforts to determine factors that influence the polar localization of the flagellum in *S. oneidensis*, we initiated this study by searching for *S. oneidensis* homologues to polar localization factors established in other bacteria, including the Tol-Pal system. During the investigation, we found that YbgC is involved in motility by regulating c-di-GMP turnover but unlikely to be associated with DSF molecules.

## Methods

### Bacterial strains, plasmids and culture conditions

All bacterial strains and plasmids used in this study were listed in Table 1. Information for primers used in this study was available upon request. For genetic manipulation, *E. coli* and *S. oneidensis* were grown in Lysogeny broth (LB, Difco, Detroit, MI) under aerobic conditions at 37 and 30 °C, respectively. When appropriate, the growth medium was supplemented with chemicals at the following final concentrations: 2, 6-diaminopimelic acid (DAP), 0.3 mM; ampicillin, 50 μg/ml; kanamycin, 50 μg/ml; gentamycin, 15 μg/ml; and streptomycin, 100 μg/ml.

**Table 1:** Strains and plasmids used in this study.

Strain or plasmid	Description	Reference or source
**Strain** ***E. coli***		
DH5α	Host for cloning	Lab stock
WM3064	Donor strain for conjugation, ∆*dapA*	W. Metcalf, UIUC
***S. oneidensis***		
MR-1	Wild type	Lab stock
FFM	∆*flaA*∆*flaB* derived from MR-1	27
HG1256	Δ*SO1256* derived from MR-1	This study
HG1856	∆*fabA* derived from MR-1	58
HG2746	∆*ybgF* derived from MR-1	This study
HG2747	∆*pal* derived from MR-1	This study
HG2748	∆*tolB* derived from MR-1	This study
HG2750	∆*tolR* derived from MR-1	This study
HG2751	∆*tolQ* derived from MR-1	This study
HG2752	∆*ybgC* derived from MR-1	This study
HG2752-1856	∆*ybgC*∆*fabA* derived from MR-1	This study
HG3210	∆*fliA* derived from MR-1	27
HG3230	∆*flrC* derived from MR-1	31
HG3231-0	∆*flrBC* derived from MR-1	31
HG3232	∆*flrA* derived from MR-1	30
HG3961	∆*rpoN* derived from MR-1	30
HG4375	Δ*SO4375* derived from MR-1	This study
**Plasmid**		
pHGM01	Ap^r^, Gm^r^, Cm^r^, *att*-based suicide vector	33
pHG101	Km^r^, promoterless broad-host vector	27
pHG102	pHG101 containing the *SoarcA* promoter	27
pHGEI01	Integrative *E. coli lacZ* reporter vector	40
pBBR-Cre	Helper vector for antibiotic marker removal	41
pHGE-P*tac*	Km^r^, IPTG-inducible P_*tac*_ expression vector	35
pET-28a(+)	His-tagged protein expression vector, Ap^r^	Novagen
pHGE-P*tac*-*ybgC*	Inducible expression of YbgC	This study
pHGE-P*tac*-*ybgC* ^*G43A*^	Inducible expression of YbgC^D15N^	This study
pHGE-P*tac*-*EcybgC*	Inducible expression of *E. coli* YbgC	This study
pHGE-P*tac*-*HiybgC*	Inducible expression of *H. influenzae* YbgC	This study
pHGE-P*tac*-*HpybgC*	Inducible expression of *H. pylori* YbgC	This study
pHGE-P*tac*-*wspR* ^*C385T*^	Inducible expression of WspR^R129C^	This study
pHGE-P*tac*-*gfp*-*ybgC*	Inducible expression of GFP-YbgC	This study
pHGE-P*tac*-*gfp*-*EcybgC*	Inducible expression of GFP-*Ec*YbgC	This study
pHGE-P*tac*-*gfp*-*HiybgC*	Inducible expression of GFP-*Hi*YbgC	This study
pHGE-P*tac*-*gfp*-*HpybgC*	Inducible expression of GFP-*Hp*YbgC	This study
pHGEI-P*ybgC-lacZ*	*E. coli lacZ* under control of *ybgC* promoter	This study
pET-*ybgC* ^WT^	pET-28a(+) expressing YbgC^WT^	This study
pET-*ybgC* ^G43A^	pET-28a(+) expressing YbgC^D15N^	This study

### In-frame mutant construction and complementation

In-frame deletion strains for *S. oneidensis* were constructed using the *att*-based fusion PCR method as described previously^33^. In brief, two fragments flanking the gene of interest were amplified by PCR, which were linked by the second round of PCR. The fusion fragments were introduced into plasmid pHGM01 by using Gateway BP clonase II enzyme mix (Invitrogen) according to the manufacturer’s instruction. Verified mutagenesis vectors were maintained in *E. coli* WM3064, which was used as the donor for subsequent conjugation, resulting in vector transfer into *S. oneidensis*. Integration of the mutagenesis constructs into the chromosome was selected by resistance to gentamycin and confirmed by PCR. Verified transconjugants were grown in LB broth in the absence of NaCl and plated on LB supplemented with 10% sucrose. Gentamycin-sensitive and sucrose-resistant colonies were screened by PCR for deletion of the target gene. Mutants were verified by sequencing the site for intended mutation.

Mutants used in previous studies have been verified by genetic complementation (Table 1). For newly constructed mutants, plasmid pHG102 was used for genetic complementation^27^. The coding sequence of the target genes was amplified and inserted into multiple cloning site of pHG102 under the control of the *S. oneidensis arcA* promoter, which is constitutively active^34^. For inducible gene expression, gene of interest generated by PCR was introduced into pHGE-P*tac* under the control of isopropyl-β-D-1-thiogalactopyranoside (IPTG)-inducible promoter P*tac* ^35^. After sequencing verification, resulting vectors were transferred into the relevant strains via conjugation for complementation and/or expression.

### Physiological characterization of *S. oneidensis* strains

Growth of *S. oneidensis* strains under aerobic or anaerobic conditions was determined by recording the optical density of cultures at 600 nm (OD_600_) and by visualizing colonies on plates. MS defined medium containing 0.02% (w/v) of vitamin free Casamino Acids was used as previously described, with 30 mM lactate as electron donor^36^. For aerobic growth, mid-log cultures were inoculated into fresh medium to an OD_600_ of ∼0.01 and shaken at 200 rpm at 30 °C. For anaerobic growth, cultures were purged with nitrogen and inoculated into fresh media prepared anaerobically to an OD_600_ of ∼0.01. The electron acceptor used in this study was fumarate at 20 mM.

Motility testing (swimming) was carried out by spotting 0.5 µl of mid-log phase (∼0.3 of OD_600_) liquid culture of *S. oneidensis* strains on LB plates with an agar concentration of 0.25% (w/v). To facilitate comparison, the wild-type strain and/or flagellin-free mutant (FFM) which is nonmotile^27^, were always included on the same plate. Photograph was taken 16 h after incubation at 30 °C unless otherwise noted. For microscopic analysis, swimming cells were scraped from the leading edges of each swarm, stained for flagellar filaments, and visualized on a glass slide with a Motic BA310 phase-contrast microscope^28^. Determination of the swimming speed of the cells in liquid media was carried out essentially as described elsewhere^37^. In brief, after ∼20 µl silicone was dropped onto a microscope slide, the cover slide (60 × 24 mm) was immediately placed on top and evenly pushed down. Slides were dried at room termperature for at least 4 h prior to use. For cell preparation, an aliquot (∼400 µl) of mid-log cultures grown in LB was placed under the cover slide of the microscopic slides and immediately analyzed microscopically. Micrographs were captured with a Moticam 2306 charged-coupled-device camera and Motic Images Advanced 3.2 software.

### GFP fusions, visualization and Western blot

To validate protein production, constructs expressing GFP fused to the C-terminal of target proteins were prepared as before^35^. After verification by sequencing, the vectors were moved into relevant *S. oneidensis* strains by conjugation. Quantitation of GFP signals was also performed^38^. In brief, mid-log phase cultures were collected, washed with phosphate-buffered saline containing 0.05% Tween 20, and resuspended in the wash buffer to an OD_600_ of ∼0.1. One hundred μl of the cell suspensions were transferred into black 96-well plates at various time intervals and fluorescence was measured using a fluorescence microplate reader (M200 Pro Tecan) with excitation at 485 nm and detection of emission at 515 nm. To determine localization of FlhF proteins, FlhF-GFP fusion proteins were used essentially the same as previously described^32^. For visualization of GFP fusions, cells were prepared with the protocol as described previously^35^. Slides were stored at 4 °C, and images were collected using a Zeiss ISM710 spectral two-photon confocal microscope.

Sodium dodecyl sulfate polyacrylamide gel electrophoresis (SDS-PAGE) and Western blotting analysis were performed as as previously described^35^. In brief, The mid-log phase cells were harvested, washed with phosphate buffered saline (PBS), resuspended in the same buffer, and subjected to SDS-PAGE (12%). After membrane transfer for 2 h at 60 V using a Criterion blotter (Bio-Rad), the blotting membrane was probed with the primary antibody Mouse Anti-eGFP-tag Monoclonal Antibody (GenScript) and then the second antibody Goat anti-Mouse IgG-HRP (Horse Radish Peroxidase) (Roche Diagnostics). Detection was performed using a chemiluminescence Western blotting kit (Roche Diagnostics) in accordance with the manufacturer’s instructions and images were visualized with the UVP Imaging System. Protein concentrations of cell lysates were determined using a Bradford assay with bovine serum albumin (BSA) as a standard (Bio-Rad).

### Flagellin extraction, purification and analysis

Flagellar filament isolation and purification was performed essentially the same as previously described^28^. In brief, 250 ml bacterial batch culture was centrifuged at 5000 *g* for 10 min at 4 °C. The cell pellet was resuspended in 10 ml PBS buffer, pH 7.0 and vortexed for 20 min to shear off flagella. The cell debris was removed by centrifugation at 10,000 *g* for 30 min at 4 °C and the supernatant containing flagellar filaments was filtered through a 0.45 μm-pore filter. The filtrate was centrifuged at 100,000 *g* for 2 h and the pellet containing concentrated filaments was resuspended in double distilled H_2_O. Protein concentrations of each sample were determined by Bradford assay using BCA as standard using the Pierce BCA protein assay kit (Thermo). Protein samples were separated by using SDS-PAGE (10%) and stained with Coomassie brilliant blue as before^28^. To determine the ratio of flagellins FlaA to FlaB, LC/MS/MS analysis of flagellins was carried out as previously described^28, 29^.

### Expression and purification of the recombinant proteins

The YbgC recombinant proteins were produced and purified to homogeneity as described before^32^. Briefly, the *ybgC* gene of *S. oneidensis* was amplified by PCR with the high-fidelity DNA polymerase Pyrobest (Takara), cloned into pET28a, in which an N-terminal six-His-tag encoding sequence was fused, and verified by sequencing. *E. coli* BL21(DE3) carrying the vector of interest was grwon in LB at 37 °C to an OD_600_ of ∼0.5 and induced by the addition of 0.1 mM IPTG for 3 to 4 h at 20 °C. The cells were harvested by centrifugation and disrupted by using a precooled French press (Constant cell disruption system, One Shot model; United Kingdom) at 18,000 lb/in^2^ for one cycle. After removal of the cell debris, the supernatant containing the His_6_-tagged recombinant proteins was purified by nickel-nitrilotriacetic acid (Ni^2+^-NTA)–agarose affinity chromatography using the purification buffers (wash buffer [50 mM NaHPO_4_, 300 mM NaCl, 40 mM imidazole, 10% glycerol] and elution buffer [50 mM NaHPO_4_, 300 mM NaCl, 250 mM imidazole, 10% glycerol]) according to the manufacturer’s instructions (GE healthcare). Imidazole and salts were then removed from the eluted fractions by overnight dialysis against 20 mM sodium phosphate buffer (pH 7.5).

### Thioesterase activity assay

The thioesterase activity of YbgC was determined by the difference in UV-visible light absorption between the substrate and the hydrolytic product as described previously^19^. In brief, hydrolysis reactions of the aryl-CoA substrates (Sigma-Aldrich) were monitored at 25 °C by recording the absorbance of 5-thio-2-nitrobenzoate at 412 nm, which was formed by 5,5′-dithio-bis-2-nitrobenzoic acid (DTNB) with CoASH released after acyl-CoA hydrolysis for 20 min. All kinetic measurements were carried out in 200 mM sodium phosphate buffer (pH 7.0) in triplicate at 25 °C. The concentration of the enzyme was adjusted to ensure that consumption of the substrate was less than 5% within the first 3 min of the reaction, during which the initial velocity (*v*) was measured. Kinetic data were collected by a Synergy 2 Multi-Detection microplate reader (M200 Pro, Tecan) and processed by GraphPad Prism. The kinetic parameters of maximum velocity (*v*_max_) and *K*_*m*_ were determined using a nonlinear regression fitting from the initial velocity data according to the Michaelis-Menten equation and the *k*_cat_ value was calculated from the ratio of *v*_max_ and the concentration of the thioesterase monomer.

### DNA synthesis and site-directed mutagenesis

The *ybgC* genes of *H. influenzae* and *H. pylori* as well as the *wspR* ^WT^ and *wspR* ^C385T^ (WspR^R129C^) genes of *Pseudomonas fluorescens* SBW25 were synthesized^39^. For site-directed mutagenesis of YbgC, residues of interest were replaced by intended ones according to the method used before^29^. Plasmid pHGE-P*tac*-*ybgC* was used as the template with a QuikChange II XL site-directed mutagenesis kit (Stratagene). Mutated PCR products were generated, subsequently digested by *Dpn*I, and transformed into *E. coli* WM3064. After sequencing verification, the resulting vectors were transferred into the relevant *S. oneidensis* strains by conjugation.

### Expression analysis

Expression of genes of interest was assessed using a single-copy integrative *lacZ* reporter system and quantitative reverse transcription PCR (qRT-PCR) as described previously^31, 40^. A fragment covering the sequence upstream of each operon tested from −400 to +1 was then amplified and cloned into the reporter vector pHGEI01, verified by sequencing, and the correct plasmid was then transferred into relevant *S. oneidensis* strains by conjugation. Once transferred into *S. oneidensis* strains, pHGEI01 containing promoter of interest integrates into the chromosome and the antibiotic marker is then removed by an established approach^41^. Cells grown to the mid-log phase under experimental settings were collected and β-galactosidase activity was measured with an assay kit and recorded with a Synergy 2 Multi-Detection microplate reader (M200 Pro, Tecan) as described previously^40^.

For qRT-PCR, total RNA was isolated from relevant *S. oneidensis* cells of mid-exponential phase using a combination of Trizon (invitrogen) with the RNeasy Mini Kit (Qiagen) and qRT-PCR analyses were carried out with an ABI7300 96-well system (Applied Biosystems) as described previously^42^. The expression of each gene was determined from three replicas in a single real-time qRT-PCR experiment. The Cycle threshold (*C*_*T*_) values for each gene of interest were averaged and normalized against the *C*_*T*_ value of the *arcA* gene, whose abundance was constant under experimental conditions^34^. Relative abundance (RA) of each gene was standardized to the *C*_*T*_ values of both the *arcA* gene using the equation RA = 2^−∆*CT*^, yielding similar fold differences.

### Determination of intracellular c-di-GMP levels

Intracellular levels of c-di-GMP were determined with LC-MS using a previously reported procedure with slight modifications^43^. Swimming cells were scraped from the leading edges of each swarm on semi-solid MS plates, suspended in lysis buffer (40% acetonitrile, 40% methanol, 0.1% formic acid), followed by 15 min of incubation on ice. Insoluble material was removed by 30,000 *g* for min at 4 °C. The resulting supernatant was collected and analyzed by using liquid chromatography-tandem mass spectrometry on an Exactive hybrid quadrupol-Orbitrap mass spectrometer (Thermo Scientific), coupled with a Thermo Accela UHPLC system. Ions were detected using multiple-reaction monitoring mode. Peaks were integrated manually in Thermo Xcalibur QualBrowser, and relative concentrations of c-di-GMP in all mutants were calculated by normalized to the average level in the wild-type.

### Bioinformatics and statistical analyses

Homologues of proteins of interest were identified via a BLASTp search of the NCBI’s nonredundant protein database, using the amino acid sequence as the query. Pairwise and multiple amino acid sequence alignments were performed by using Clustal Omega program^44^. The relative intensity of specific protein signals on SDS-PAGE was measured using ImageJ^45^. Student’s *t* test was performed for pairwise comparisons. Statistical analysis tools integrated in Excel were used to determine correlation of data sets with coefficient of determination (R^2^) and polynomial regression. Values were presented as means +/− standard error (SE).

## Results

### *S. oneidensis* has a Tol-Pal but lacks a counterpart for *Pa*Poc

In addition to the Tol-Pal system, two other systems recently have been proposed to play a critical role in regulation of flagellum polarity in polarly flagellated bacteria^46, 47^. One is HubP of *Vibrio cholerae*, which is polarly localized and functions to recruit other polarly posited proteins, including FlhF^48^. However, functional orthologs (SO_3069 and Sputcn32_2422) of *Vc*HubP in *Shewanella* are dispensable for flagellar positioning at the pole although it is crucial for normal flagellar function^49^. The other is the Poc complex (TonB3-PocA-PocB) of *Pseudomonas aeruginosa*, which plays an essential role in coordinating both polarly located pilus and flagellum^50^. To test whether *S. oneidensis* possesses counterparts of *E. coli* Tol-Pal and *Pa*Poc, we performed a BLASTp search against the *S. oneidensis* proteome. Clearly, *S. oneidensis* possesses a complete Tol-Pal system (Fig. 1). In the case of *Pa*Poc, multiple putative homologues were found (Table S1). Based on E-value, protein size, and synteny, among the homologous proteins of *Pa*PocA, it is apparent that *So*TolQ is the most likely analogue. More importantly, *So*TolR is the only protein that is homologous to *Pa*PocB. Hence, we propose that *S. oneidensis* possesses a Tol-Pal complex, but may not have a counterpart of *Pa*Poc.

### *So*YbgC has a role in motility

**Figure 2:**
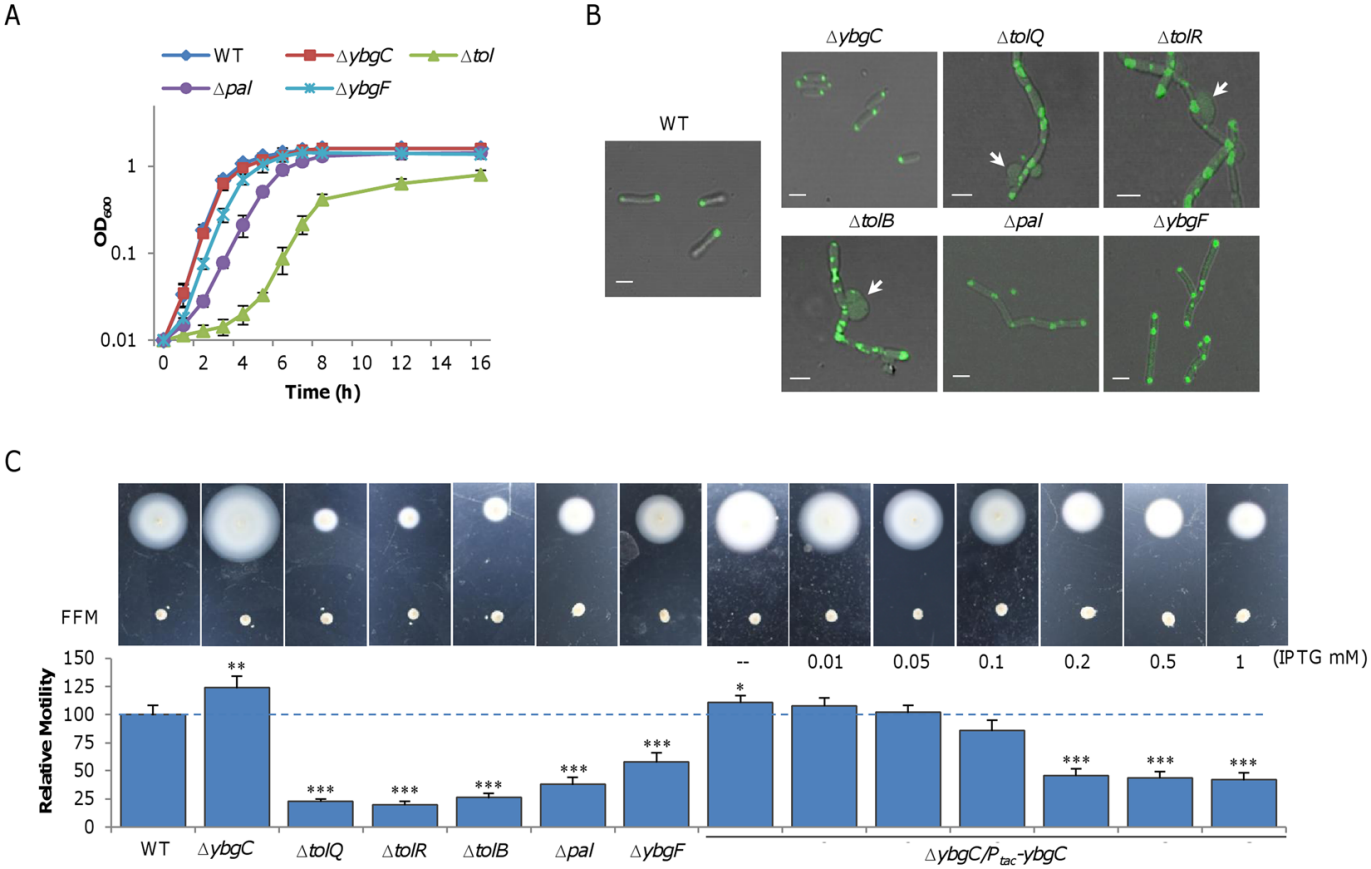
Characteristics of *S. oneidensis* mutants for genes in the *tol*-*pal* cluster. (**A**) Growth under aerobic conditions. Shown were wild-type (WT) and its isogenic mutants. The fresh medium was inoculated with mid-log phase cultures (∼0.3 of OD_600_) for each strain. Both LB (shown) and MS defined medium were used and similar results were obtained. ∆*tol* represents all *tol* mutants (∆*tolQ*, ∆*tolR*, and ∆*tolB*) because growth defects in these strains were similar. (**B**) Morphology of cells expressing FlhF-GFP. Cells of indicated strains grown to the mid-log phase in LB broth were examined for morphological phenotype and FlhF localization with a confocal microscope. Arrows highlight clear examples of blebs found in *tol* mutants but not other mutants. The scale bar represents 1 µm in all panels. (**C**) Motility on the semi-solid agar plates. Strains indicated were grown to the mid-log phase were spotted on plates along with nonmotile FFM (flagellin-free mutant) and incubated for 16 h. Complementation of the ∆*ybgC* strain by inducible expression from pHGE-P*tac* was performed with IPTG ranging from 0 to 1 mM. Relative motility for each mutant was given by setting the motility of WT (the diameter) as 100%. Asterisks indicate statistically significant differences (*P < 0.05; **P < 0.01; ***P < 0.001; *n* ≥ 3). All experiments were performed at least three times with either representative results shown or with the standard error of the mean (SEM) presented as error bars.

Tol-Pal appears to be only known system that may be involved in regulating the polar localization of the flagellum in *S. oneidensis* ^4, 9^. To test this, attempts were made to construct In-frame deletion mutants for each gene within the *tol-pal* cluster. Knock-out mutants for all genes except *tolA* were obtained, implying that TolA is probably essential to *S. oneidensis*. With respect to growth, all of resulting mutants but ∆*SoybgC* displayed significantly reduced rate compared with the wild-type (Fig. 2A). Depletion of each of three Tol proteins (TolQ, TolR, and TolB) had effects more substantial than the lack of Pal or YbgF. Importantly, the defects are due to the mutations because cells largely restored normal growth when the respective genes were expressed *in trans* (Fig. S1).

A common phenotype caused by depletion of one or more components of the Tol-Pal system is that cells grow into cell-chain. Microscopic analysis of these *S. oneidensis* mutants revealed a similar phenotype, albeit varying in degree on individual mutations and *tol* mutants being more drastic (longer chain) (Fig. 2B). A more substantial difference found between the *tol* and *pal* (and *ybgF*) mutants was the frequent presence of big blebs at the cell surface of the former, implicating a defect in PG^7^. In line with the failure in construction of *tolA* knockout, these results conclude that Tol proteins are more critical to *S. oneidensis* cell morphology. Again, the *SoybgC* mutant was indistinguishable from the wild-type in these aspects. In addition, we found that FlhF-GFP fusions were mainly located to the cell pole in both the wild-type and ∆*SoybgC* strains (Fig. 2B). Despite morphological differences, the fusions in all other mutants were found mainly at the cell pole and/or division sites of the cell chain, suggesting that neither YbgC nor the Tol-Pal system affects FlhF localization. Similar results were obtained from these mutants without carrying the vector expressing FlhF-GFP fusions (Fig. S2).

In the case of motility, all mutants with growth defect displayed heavily impaired ability to move on soft agar plates (Fig. 2C). By comparing to the flagellin-free mutant (Δ*flaAB*), however, none of mutations completely abolishes motility. Owing to the growth and division defects, whether the reduced motilities associate with the flagellar system is difficult to assess. In contrast, the Δ*SoybgC* strain clearly had enhanced motility (Fig. 2C). To confirm this, we placed the gene under the control of IPTG-inducible promoter P*tac* and assessed effects of its expression at varying levels on motility^35^. Because P*tac* is slightly leaky^31, 51^ (Fig. S2), in the absence of IPTG the Δ*SoybgC* strain carrying the cloned gene displayed a significant reduction in motility (Fig. 2C). Expression of the gene at 0.05 mM IPTG restored motility to the wild-type level. Consistently, we found that the *SoybgC* promoter activity was comparable to that of P*tac* at 0.05 mM IPTG by using a *lacZ* reporter (Fig. S3A). Motility decreased with IPTG concentrations up to 0.2 mM inversely, not necessarily in a proportional manner. But this phenomenon disappeared in the presence of IPTG at further increased levels, possibly due to that the effect of the mutation is saturated. In the case of growth, YbgC in excess did not elicit significant difference compared to the wild-type (Fig. S3B), excluding the possibility that the reduction in motility is a result of retarded growth. Based on all of these data, we conclude that *So*YbgC is involved in motility.

### Loss of *So*YbgC enhances motile capacity of individual cells

**Figure 3:**
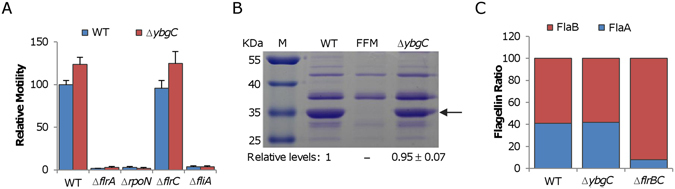
*S. oneidensis ybgC* mutant assembles a normal flagellum. (**A**) The *ybgC* mutation did not interfere with regulation of flagellar assembly. Four regulators were under test. Please note that FlrC is not essential to flagellar assembly in *S. oneidensis*. (**B**) Estimation of the flagellar length with SDS-PAGE analysis of isolated flagellins, whose levels are proportional to flagellar filament length. Flagellins from the same volume of mid-log phase cultures (adjusted to the same optical density) were extracted and separated on SDS-PAGE. The band intensity was estimated by using ImageJ. Relative flagellin levels were calculated by normalizing to the average level of WT, which was set to 1 for presentation. (**C**) Estimation of ratio of flagellin FlaA to FlaB with LC-MS/MS analysis of flagellins obtained in B. Aliquots of flagellins were trypsin-digested and analyzed by LC-MS/MS to determine relative abundance of flagellins FlaA and FlaB. All experiments were performed at least three times with either representative results shown or with SEM presented as error bars.

Discovery of an unexpected motility phenotype raised an interesting question about the role played by *So*YbgC in *S. oneidensis*. We have previously illustrated that the flagellar assembly in *S. oneidensis* is governed by an atypical four-tier regulatory system, which differs from that of *V. cholerea* in that the *S. oneidensis* FlrBC regulatory system is not essential to motility^31^. To determine if *So*YbgC interferes with the flagellar assembly, we removed the *SoybgC* gene from strains lacking one of the flagellar regulators, including Δ*flrA*, Δ*rpoN*, Δ*flrC*, and Δ*fliA*. In the case of regulators that are essential to motility, the additional removal had no effect on motility of these mutants (Fig. 3A). In contrast, in the absence of FlrC, the *So*YbgC depletion resulted in enhanced motilities, that are comparable to those of the ∆*SoybgC* strain (Fig. 3A). Moreover, none of flagellar regulators was found to mediate expression of *SoybgC* (Fig. S3A). These data, collectively, imply that *So*YbgC may not play a role in the flagellar assembly.

To further investigate the possibility that the hypermotility resulting from the *So*YbgC loss is due to changes in the flagellum *per se*, we examined other flagellar factors that impact motility in *S. oneidensis*. In the wild-type population grown at the edge of bacterial swarms on semi-solid agar LB plates, approximately 60% were flagellated (Table 2). This percentage is in excellent agreement with the results of previous studies^28, 37^. In the Δ*SoybgC* population, a similar portion of cells possessed a flagellum, supporting that the flagellar assembly is not affected by the mutation as suggested above. We then compared the swimming speed of individual cells between the wild-type and Δ*SoybgC* strains (Table 2). While cells of the wild-type were monitored to swim at ∼53 µm per second, Δ*SoybgC* cells revealed an increase in swimming rate to ∼68 µm per second, approximately 130% relative to the wild-type, suggesting that the mutation enhances locomotive capacity of individual cells.

**Table 2:** Flagellar characteristics of *S. oneidensis ybgC* mutant.

Strain	Motility (%)	Flagellated (%)	Swimming speed (µms^-1^)^a^
WT	100	57 ± 6	53 ± 12
FFM	0	0	0
∆*ybgC*	124 ± 7	54 ± 9	68 ± 19
∆*ybgC/ybgC* ^WT^	98 ± 6	53 ± 8	50 ± 11
∆*ybgC/ybgC* ^D15N^	126 ± 11	59 ± 4	70 ± 17

^a^A minimum of 100 cells were measured.

In bacteria with a single polar flagellum, the length, composition, and rotation rate of the filament dictate motility. Length of filaments was estimated by SDS-PAGE analysis of flagellin quantities from cells of the similar numbers. Evidently, comparable amounts of flagellins were produced by both the wild-type and Δ*SoybgC* strains (Fig. 3B), indicating filament length unlikely a critical factor in explaining the motility difference. Furthermore, we examined the ratio of two flagellins, FlaA and FlaB, a factor which also plays an important role in motility^31^. This is because, with respect to motility, FlaB is predominant but effect of FlaA is negligible^27, 29^. The consequence is that motility increases with the ratio of FlaB to FlaA^31^. By using LC/MS/MS, we found that the ratio of two flagellins, based on averaged intensities of unique signature peptides for each flagellin, was not significantly affected by the *SoybgC* mutation (Fig. 3C). These observations thus manifest that neither the filament length nor the composition is critically altered in the Δ*SoybgC* strain. Thus, we propose that the rotation rate of the filament is likely accountable for the hypermotility of the *SoybgC* mutant although we were unable to accurately measure it.

### Thioesterase activity is required for the role of *So*YbgC in motility

**Figure 4:**
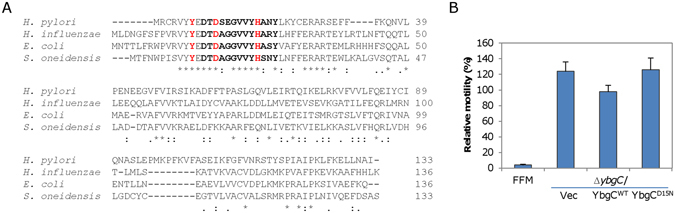
Thioesterase activity is required for the role of SoYbgC in motility. (**A**) Sequence alignment of *So*YbgC with YbgC proteins from *E. coli*, *H. influenzae*, and *H. pylori*, whose thioesterase activity has been confirmed. The consensus sequence for the active site core of thioesterase is in bold. Three residues that are essential to thioesterase activity are in red, of which Asp15 is subjected to mutation to create mutants deficient in thioesterase activity. (**B**) Effect of *So*YbgC^D15N^ on motility. Expressed *So*YbgC^D15N^ in the ∆*ybgC* strain under P*tac* in the presence of 0.05 mM IPTG could not function as the wild-type with respect to motility. Vec and YbgC^WT^ represent the empty plasmid and the vector carrying the wild-type YbgC, respectively. The experiment was performed at least three times with SEM presented as error bars.

*S. oneidensis* YbgC is annotated to be a thioesterase and its counterparts in bacteria, such as *E. coli*, *H. influenzae*, and *H. pylori*, show thioesterase activity although their substrates differ^17, 19, 52^. A sequence analysis revealed a comfortable sequence similarity (against *Ec*YbgC, E-value, 5e-40) between *S. oneidensis* YbgC and those whose thioesterase activity had been established (Fig. 4A). However, it shares the consensus sequence [DTD-X_(2)_-GVV-X-H-X_(2)_-Y] that defines the active site core^53^, suggesting that the protein likely has thioesterase activity. To test this, we overproduced the recombinant *So*YbgC with the 6x His-tag at the N-terminus in *E. coli* and purified it to homogeneity (Fig. S4). Various acyl-CoAs were used as the substrates in thioesterase activity assay with *So*YbgC and DTNB. As shown in Table 3, *So*YbgC apparently had a preferred activity for short-chain acyl-CoA substrates such as acetyl-CoA and propionyl-CoA. Thioesterase activity of the enzyme reduced with length of acyl-CoA substrates; both butyryl-CoA and octanoyl-CoA were consumable substrates. In contrast, no activity was detected for lauroyl-CoA or palmitoyl-CoA, indicating that *So*YbgC does not work with acyl-CoA substrates that have 12 carbons or more. These data confirm that *So*YbgC is an enzyme with thioesterase activity.

**Table 3:** Kinetic constants for *So*YbgC-catalyzed hydrolysis of acyl-CoAs at pH 7.5 and 25 °C.

Acyl-CoA substrate	*K*_*m*_ (µM)	*k*_*cat*_ (s^−1^)	*k*_*cat*_/*K*_*m*_ (s^−1^ × M^−1^)
Acetyl-CoA	3.5 ± 0.4	8 ± 0.7	2.3 × 10^6^
Acetyl-CoA^a^	231 ± 37	0.068 ± 0.01	2.9 × 10^1^
Propionyl-CoA	4.8 ± 0.6	3.9 ± 5	8.1 × 10^5^
n-Butyryl-CoA	17 ± 2	1.3 ± 0.1	7.6 × 10^4^
Octanoyl-CoA	34 ± 4	0.15 ± 0.01	4.4 × 10^3^
Lauroyl-CoA	—	<10^−4^	—
Palmitoyl-CoA	—	<10^−4^	—

^a^Performed with *So*YbgC^D15N^ mutant.

To determine whether thioesterase activity of *So*YbgC is required for motility regulation, we made attempts to express mutant proteins whose thioesterase activity is abolished. According to reports on other YbgC proteins^16^, Tyr11 and the active site core residue Asp15 and His22 in *So*YbgC are crucial residues for hydrolysis of acyl-CoA substrates (Fig. 4A). In particular, the essentiality of Asp15 to thioesterase activity has been firmly established on the findings that the replacement of the Asp15 counterpart by Asn inactivates such enzymes, such as *Hi*YbgC and a *Pseudomonas* thioesterase^17, 54^. Hence, we performed site-directed mutagenesis for generating *So*YbgC^D15N^. In the Δ*ybgC* strain, production of the resulting mutant proteins was driven by the P*tac* promoter as for the wild-type *So*YbgC used above. In the presence of 0.05 mM IPTG, a concentration at which the wild-type motility was restored with the wild-type *So*YbgC, the Δ*ybgC* strain producing *So*YbgC^D15N^ remained similarly hypermotile (Fig. 4B). To confirm that the mutant protein loses thioesterase activity, we purified *So*YbgC^D15N^ in the same manner as *So*YbgC and found that it was virtually unable to catalyze the hydrolysis of acetyl-CoA (Table 3). Based on these data, we conclude that thioesterase activity is essential to the regulatory role of *So*YbgC in motility.

### Regulation of motility by YbgC proteins is not universal

**Figure 5:**
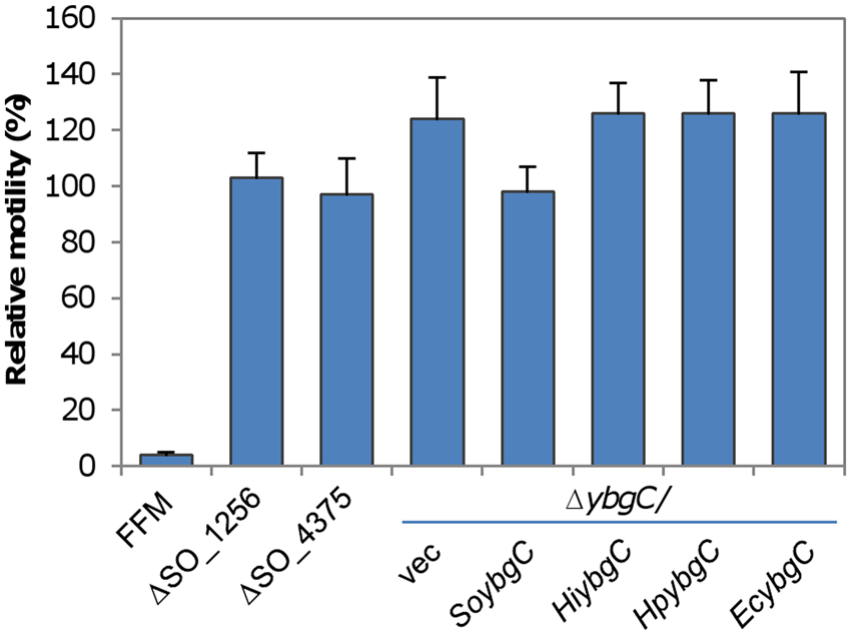
Unique features of SoYbgC may account for its regulatory role in motility. Effect of various thioesterases on motility. Various thioestereases, including YbgC proteins from *E. coli*, *H. influenzae*, and *H. pylori*, under P*tac* were expressed in the ∆*ybgC* strain in the presence of 0.05 mM IPTG. The experiment was performed at least three times with SEM presented as error bars.

To date, this is the first report that suggests a link between an YbgC thioesterase and motility. There are two other YbgC-family thioesterases encoded in the genome, SO_1256 (137 a.a.) and SO_4375 (144 a.a.), which are similar in length and share modest sequence similarities to *So*YbgC (E-value of BLASTp, 9e-13 and 4e-11, respectively) (Fig. S5). To determine whether the hypermotility phenotype of the ∆So*ybgC* strain is attributable to any YbgC-family thioesterase, we constructed strains lacking either SO_1256 or SO_4375 and examined their motility. Clearly, difference in motilities of these two mutants and the wild-type was insignificant (Fig. 5), manifesting that *So*YbgC is the only member of YbgC-family thioesterases that participates in motility regulation.

We next addressed whether YbgC proteins in other bacteria can function as *So*YbgC with respect to motility regulation. To this end, we performed heterogeneous complementation with *Hi*YbgC, *Hp*YbgC, and *Ec*YbgC as we did with *So*YbgC, all of which are proven to have thioesterase activity^16^. Production of all these three proteins was confirmed by using GFP fusion and GFP fusions were likely to be functional because GFP-*So*YbgC complemented the phenotype of ∆*SoybgC* as effective as *So*YbgC (Fig. S6). In presence of 0.05 mM IPTG, none showed any effect on motility of the Δ*ybgC* strain (Fig. 5), ruling out the possibility that these YbgC proteins are able to regulate motility. All together, these findings implicate that *So*YbgC has some unique characteristics that influence motility.

### The *SoybgC* mutation reduces intracellular c-di-GMP levels

Thioesterases are essential in biosynthesis of diffusible signal factor (DSF), which contributes to bacterial virulence, formation of biofilms, antibiotic tolerance, and various types of locomotion^55, 56^. To test whether DSF factors are associated with the pheontype caused by the *ybgC* mutation in *S. oneidensis*, we examined relevant processes^57, 58^; based on the results (Fig. S7), we propose that *S. oneidensis* is unlikely to produce DSF molecules.

**Figure 6:**
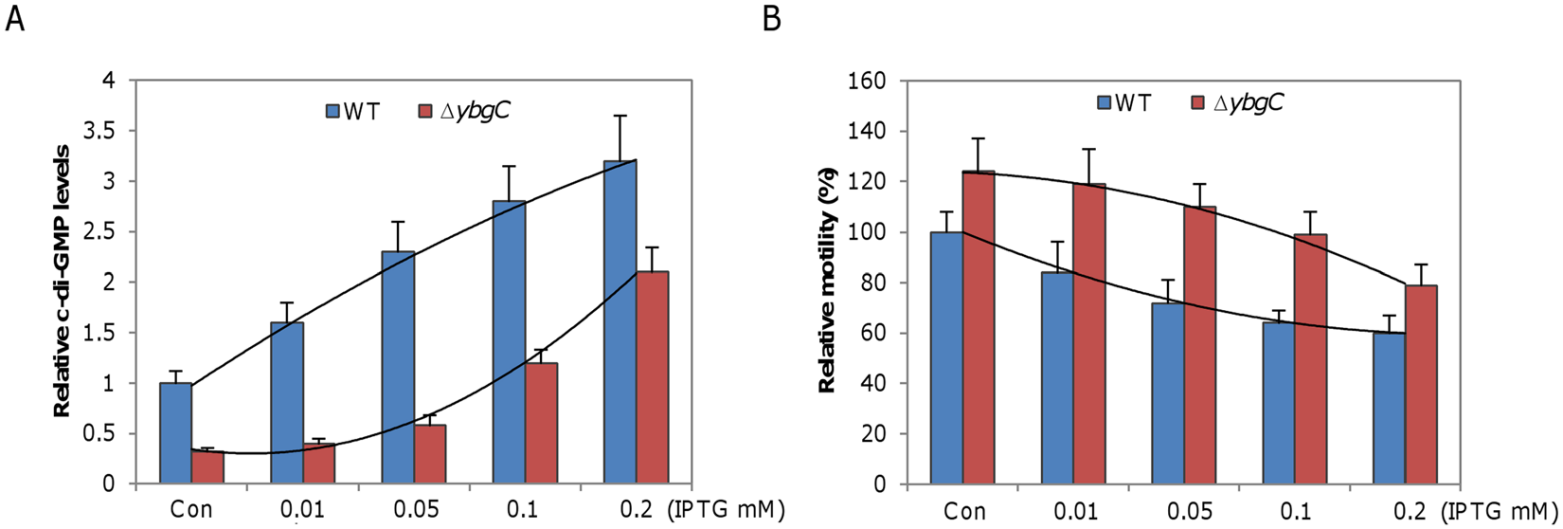
*So*YbgC-mediated regulation of motility functions through c-di-GMP. (**A**) Relative intracellular levels of c-di-GMP measured by LC-MS/MS. *P. fluorescens* WspR^R129C^, a constitutive active c-di-GMP synthetase, under P*tac* was expressed in the wild-type and ∆*ybgC* strains in the presence of IPTG at indicated levels. The levels of c-di-GMP in WT carrying the empty vector were set to 1. (**B**) Relative motility. Motilities of the strains cultivated under the same condition as in A were compared. In both **A** and **B**, a second-order polynomial best fit for each strain is given and con represents the experimental condition that both strains carry the empty vector.

Second messenger molecule c-di-GMP controls a variety of cellular processes, including motility, to mediate transition between motile and sedentary forms of bacterial life^21^. Recent studies have linked c-di-GMP to thioesterases via new sensor proteins for perception of DSF-family signals that modulates c-di-GMP turnover^23, 59^. Despite the lack of DSF in *S. oneidensis*, these findings motivated us to look for a possible relationship between *So*YbgC and intracellular c-di-GMP levels in *S. oneidensis*. Intracellular levels of c-di-GMP were measured by LC/MS-MS. Depletion of *So*YbgC caused a substantial decrease in the intracellular c-di-GMP concentration, only ∼30% relative to that of the wild-type (Fig. 6A). When the *SoybgC* gene was expressed *in trans*, c-di-GMP levels increased with IPTG concentrations, but this effect was not observed in the Δ*ybgC* strain producing *So*YbgC^D15N^ (Fig. S8). These data indicate that *So*YbgC mediates c-di-GMP homeostasis and thioesterase activity is essential to this role.

To confirm that c-di-GMP is responsible for the hypermotility phenotype of the *SoybgC* mutant, we manipulated intracellular c-di-GMP levels by producing *Pseudomonas fluorescens* WspR^R129C^ to varying levels^39^. WspR^R129C^, a mutant of WspR which is a diguanylate cyclase, is constitutively active. When produced, WspR^R129C^ increased intracellular c-di-GMP concentrations in both the wild-type and ∆*SoybgC* strains with IPTG (Fig. 6A). However, R^2^ (coefficient of determination) values for the wild-type and ∆*SoybgC* strains were 0.98 and 0.85, respectively. This difference suggests that factors other than YbgC are also involved in c-di-GMP homeostasis under experimental conditions. In line with this, the responding patterns of c-di-GMP concentrations to IPTG levels, based on polynomial regression, between these two strains were also apparently different. In the wild-type, c-di-GMP concentrations increased with IPTG (up to 0.2 mM) nearly in a linear manner. On the contrary, effects of WspR^R129C^ on c-di-GMP concentrations in the ∆*ybgC* strain were much more drastic with IPTG at relatively high levels (0.1 and 0.2 mM) than those with low levels (no more than 0.05 mM), implying that depletion of YbgC counteracts the activity of WspR^R129C^. In the case of motility, expression of WspR^R129C^ reduced motilities of the wild-type and ∆*SoybgC* strains with IPTG levels (Fig. 6B), a scenario consistent with the notion that c-di-GMP inhibits motility^60^. Evidently, the *SoybgC* mutant was more resistant to WspR^R129C^ overproduction, especially when IPTG levels were low. These data, collectively, conclude that the *SoybgC* mutation enhances c-di-GMP degradation in *S. oneidensis*.

## Discussion

Thioesterases catalyze hydrolysis of the thioester bond between a carbonyl group and a sulfur atom^16^. As thioesters are widely found in a variety of metabolites from numerous biological processes, thioesterases play a critical role in metabolism, membrane biosynthesis, signal transduction, and gene regulation^16^. Based on enzyme function and substrate specificities, thioesterases are grouped into 23 families almost unrelated to one another by primary structure, of which YbgC and YbgC-like proteins constitute number nine, namely TE9^16^. The majority of thioesterases, including YbgC, have a hot-dog fold, whose signature is a five-stranded antiparallel β–sheet around an elongated α–helix^61^.

A recent report about the *E. coli* Tol-Pal system has revealed that YbgF coordinates envelope machines facilitating septal PG synthesis and OM constriction (Tol system), leaving YbgC the only protein encoded in the *tol*-*pal* cluster without a clearly defined role^12^. Compared to established Tol-Pal members, YbgC bears two significant differences. First, YbgC is located in the cytoplasm, contrasting IM-associated TolA, TolQ, and TolR, OM-associated Pal, and soluble TolB and YbgF in the periplasm^10, 12^; hence it may not directly work with these Tol-Pal members at the IM constriction site of the periplasmic side. Second, in certain bacteria the *ybgC* gene is missing in the *tol*-*pal* cluster^12^. Proteins in the hotdog fold superfamily are characterized by the low sequence homology but the same structural fold, leading to a weak correlation between the sequence similarity and protein function^53^. Take *E. coli* as an example. Although its YbgC is the closest homologue to a cyanobacterial hotdog fold thioesterase involved in phylloquinone biosynthesis, it does not play an equivalent role. Rather, YdiI, one of eight other hotdog fold thioesterases, performs the function^52^. In addition, EntH and YdiI, two hotdog fold thioesterases highly homologous to each other (E-value, 5e-48; identity, 60%), carry out completely different physiological roles^52^. As a consequence, whether a genuine *ybgC* gene is present in the bacteria with an *ybgC*-free *tol-pal* cluster remains enigmatic.

YbgC proteins, found only in bacteria, have acyl-CoA hydrolase activity^16^. *S. oneidensis* YbgC, as revealed in this study, hydrolyze primarily short-chain acyl-CoA thioesters. While YbgCs with a similar preference have been reported, others favor long-chain acyl-CoA thioesters^17, 19^. However, there is a caveat for the statement: all thioesterases under examination are genuine YbgC. *So*YbgC and *Hi*YbgC, representatives for the former, are no doubt YbgC proteins because their coding genes are clustered with the *tol*-*pal* genes^17^. The identity of *Hp*YbgC, a representative for the latter, remains uncertain as it is not associated with the *tol*-*pal* genes^19^.

Although YbgC proteins are firmly established to be thioesterase, their cellular role of YbgC proteins has remained elusive. We showed that *S. oneidensis* YbgC is linked to motility, a phenomenon relying on its thioesterase activity. None of *Hi*YbgC, *Ec*YbgC, and *Hp*YbgC is able to complement the *So*YbgC loss. The failure with *Hp*YbgC is reasonable because their substrates are distinct, but the same result with *Hi*YbgC is unexpected. However, this provides further evidence to support the characteristics of hotdog fold family proteins, that is, a weak correlation between the sequence similarity and protein function^53^. It is worth mentioning that *So*YbgC, as well as the Tol-Pal proteins, are not required for flagellar positioning.

Proteins possessing thioesterease activity that have been previously implicated a role in motility are those responsible for DSF generation, including RpfF of some bacteria such as *Xanthomonas* and DfsA of *B. cepacia* that are active with acyl-ACP rather than acyl-CoA^25, 62^. Based on the lack of homologue to RpfF or DfsA, the negligible role of the spent medium from a *So*YbgC overproducing strain on motility, and the irrelevance of a *SofabA* mutation in the phenotype of the *SoybgC* mutant, we propose that *S. oneidensis* is unlikely to produce DSF molecules.

Enhanced motility of the *SoybgC* mutant is attributed to reduced c-di-GMP levels. In addition, the mutation counteracts the effects of expressed c-di-GMP synthetase, especially at low amounts. It is therefore conceivable that the *SoybgC* mutant has a stronger c-di-GMP degradation capability. Cyclic di-GMP is synthesized by diguanylate cyclases, characterized by a canonical GGDEF motif and hydrolyzed by phosphodiesterases, characterized by conserved EAL or HD-GYP motifs, respectively^21^. *S. oneidensis* is renowned for its large repertoire of proteins involved in c-di-GMP turnover and signaling, including 51 diguanylate cylases, 27 phophodiesterases, and 20 hybrid diguanylate cylase or phophodiesterase proteins^24^. Many of these proteins contain additional domains, such as Che, Per-Arnt-Sim (PAS), and NIT domains, which are important signaling modules shown to respond to various environmental and cellular cues^60, 63^. For example, the PAS domain of *Burkholderia cenocepacia* RpfR, a hybrid protein with domains organization of PAS-GGDEF-EAL, accounts for sensing a DSF family signal and subsequently mediates c-di-GMP turnover^23^. We expect one or some of these proteins may link the YbgC function with c-di-GMP. Efforts to test this notion are under way.

## Electronic supplementary material

All supplemental materials
