# Supplementary material for: Thioesterase YbgC affects motility by modulating c-di-GMP levels in Shewanella oneidensis
Source: Sci Rep. 2017 Jun 21;7:3932. doi: 10.1038/s41598-017-04285-5 (PMC5479800; doi:10.1038/s41598-017-04285-5)
Supplement: Supplementary file 1 — All supplemental materials [file 41598_2017_4285_MOESM1_ESM.pdf]

Supplemental materials of

Thioesterase YbgC affects motility by modulating c-di-GMP levels in *Shewanella oneidensis*

Tong Gao, Qiu Meng, Haichun Gao*

Institute of Microbiology and College of Life Sciences, Zhejiang University, Hangzhou, Zhejiang, 310058, China

TABLE S1. *S. oneidensis* homologues of *P. aeruginosa* Poc components

P. aeruginosa	*S. oneidensis*	Length (a.a.)	E-value	Predicted function in *S. oneidensis*
PocA (211 a.a.)	TtpC (SO1825)	451	2e-15	TonB2 energy transduction system inner membrane component TtpC
	TolQ (SO2751)	228	2e-11	TolA energy-transducing system inner membrane component TolQ
	ExbB1(SO3671)	260	2e-11	TonB1 energy transduction system for heme uptake inner membrane component ExbB
	ExbB2(SO1826)	160	1e-10	TonB2 energy transduction system inner membrane component TtpC
PocB (146 a.a.)	TolR (SO2750)	144	0.006	TolA energy-transducing system inner membrane component TolR
TonB3 (319 a.a.)	TonB1(SO3670)	274	3e-04	TonB1 energy transduction system for heme uptake energy transducer component TonB
	SO2785	1088	8e-04	ribonuclease E, Rne
	SO4110	429	0.29	MSHA biogenesis protein MshN
	SO4564	203	0.65	TonB2 protein

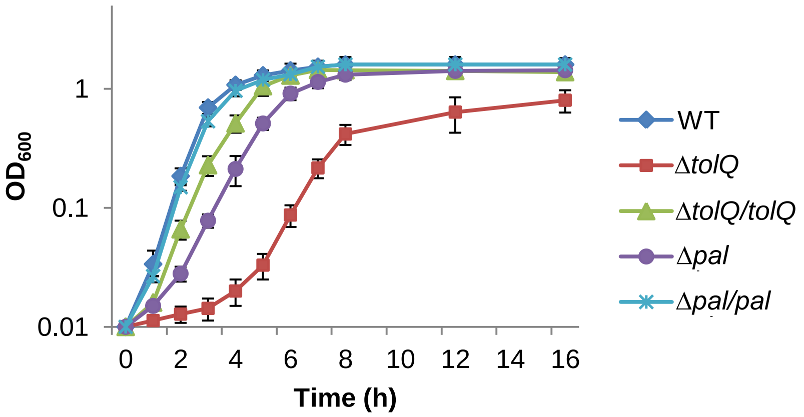

FIG S1. Complementation of *S. oneidensis* mutants for genes in the *tol-pal* cluster with respect to growth. Growth conditions were the same as Fig. 2A. Shown were wild-type (WT) and its isogenic mutants without or with respective genes within pHG102. Expression of indicated genes was controlled by the *S. oneidensis arcA* promoter, which is constitutively active. The *pal* gene expression *in trans* fully restored growth to the wild-type level. However, the *tolQ* gene substantially, but not fully, improved growth. This may be due to difference in expression levels of *tolQ* in the wild-type and in the complemented strain. All experiments were performed at least three times with the standard error of the mean presented as error bars.

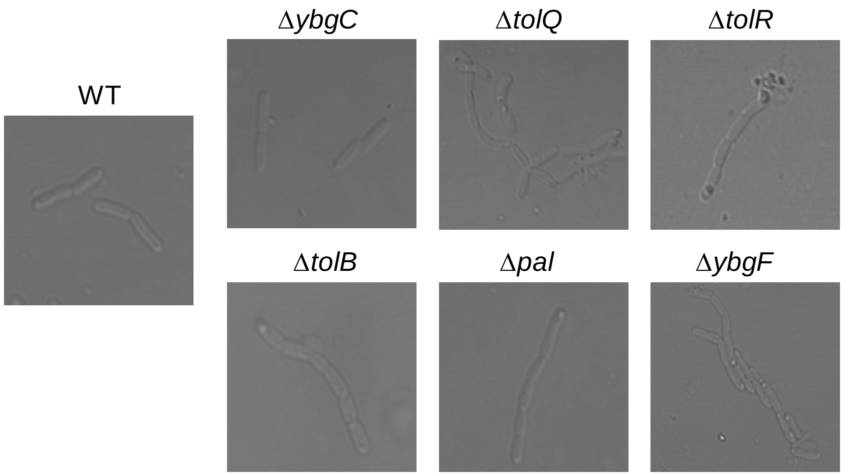

FIG. S2. Morphology of *tol-pal* mutant cells. Cells of indicated strains grown to the mid-log phase in LB broth were examined for morphological phenotype with a phase-contrast microscope.

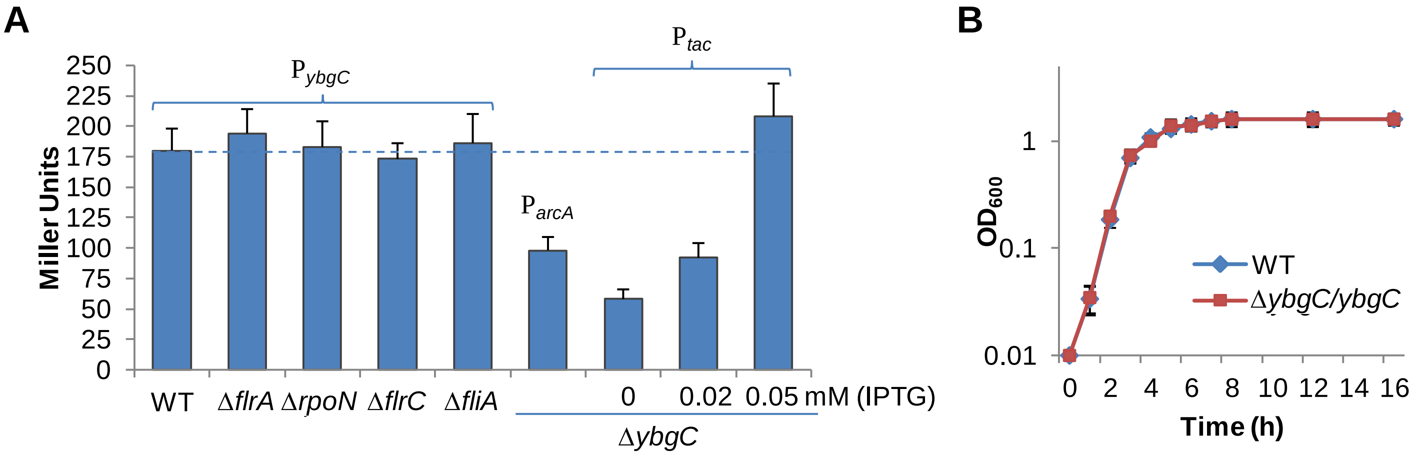

FIG S3. A. Calibration of promoters used in this study. Indicated promoters were cloned into a *lacZ* reporter and their activity in mid-log phase cells was assayed. For P*arcA* and P*tac*, the Δ*ybgC* strain was used as the test host. The promoter of the *S. oneidensis arcA* gene, which is relatively stable, was included as a control. **B.** Growth of the Δ*ybgC* strain overexpressing *ybgC* under aerobic conditions. Shown were wild-type (WT) and the Δ*ybgC* strain in the presence of 1 mM IPTG. Error bars represent the standard error of the mean from at least three independent experiments.

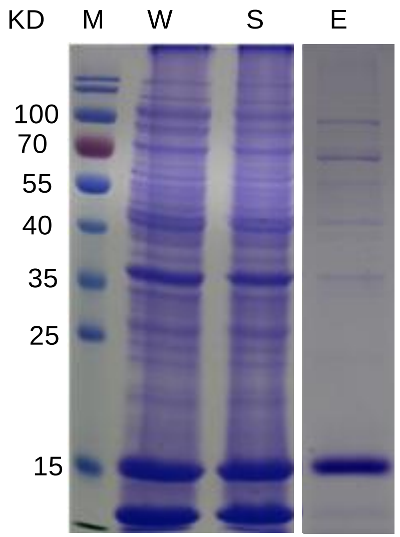

FIG S4. SDS-PAGE analysis of over-expressed recombinant YbgC protein. M, molecular mass marker; W, crude extract of *E. coli* BL21(DE3); S, supernatant of disrupted cells; E, purified recombinant YbgC protein.

```
YbgC             -----MTFNWPISVYYEDTDAGGVVYHSNYLNFFERARTEWLKALGVSQTALLADDTAFVVKRAELDFKKAARFEQ
SO_1256          MPQFSPSYSFPVQIYYEDTDFSGVVYHPNFLKYFERAREHVIGAEQLRNL-WQQQQLGFAVYRSDMLCHEGVEFAD
SO_4375          -MKALLSIEMDMQVPFHDVDSMGITWHGNYLRYFEVARCKLLDELGYNYRQMRASNYAWPIIDVQIKYVKPSTFEQ
                       : .  :.: :.*.*  *:.:* *:*.:** ** . :            .: .: :   ::   :   * :

YbgC             NLIVETKVIELKKASLVFHQRLVDHLG----DCYC-EGTVLV----ACVALSRM---RPRAIPLNIVQEFDSAS
SO_1256          IIDIRTKFYFESKYRTVWHQEIWRPNG----KKPAVTATIEM----VCMNQARQ---L-APMPAELITQLSLGM
SO_4375          HITVRAELVEWENRLKINYQIRDTATGERITKGYTIQAAVDMTSQEMCFVTPEVFRSKIAPLLAKVDNE-----
                  : :.::.   .:   : :*      *    .     .:: :     *.   .        :  ::  :
```

FIG S5. Sequence alignment of YbgC and other two hot-dog fold thioestereases *in S. oneidensis*. Residues for catalytic active sites are in red.

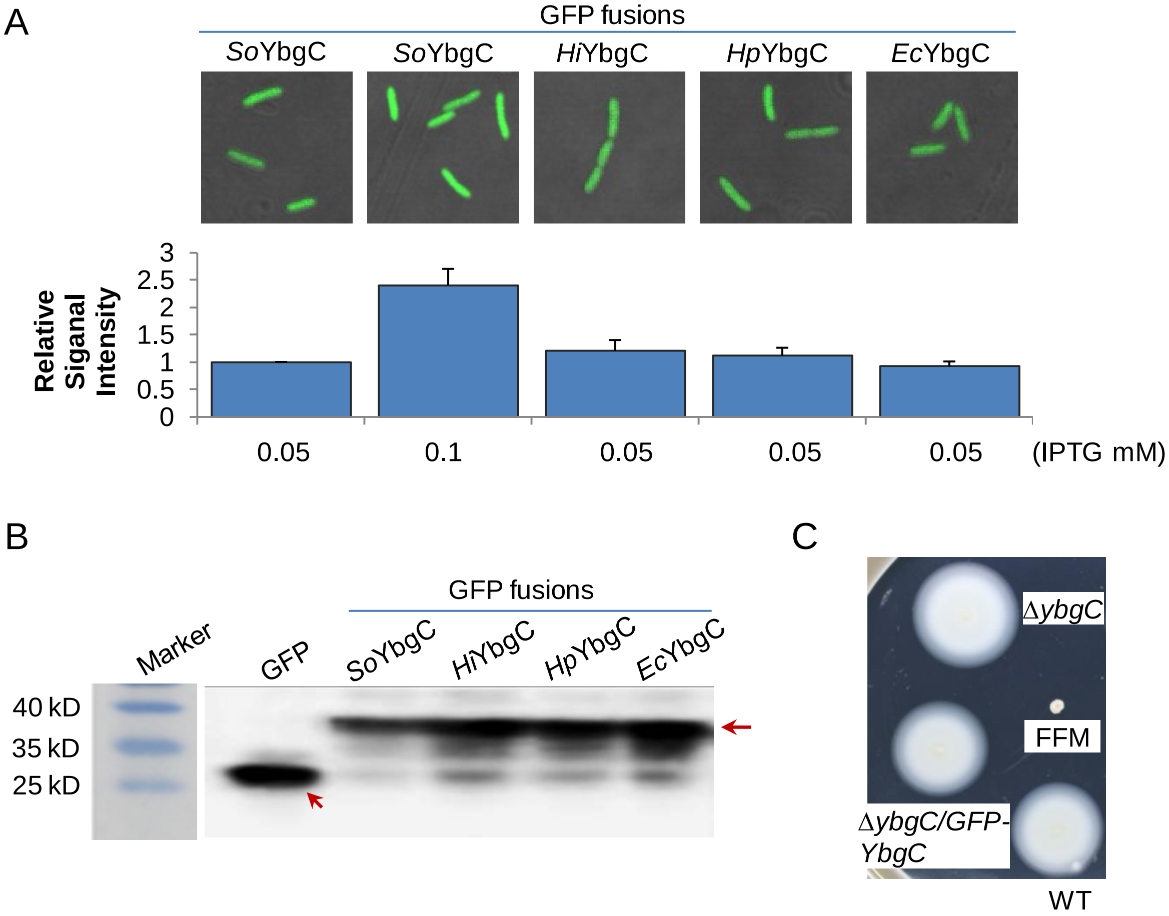

FIG S6. GFP-YbgC fusion proteins. **A.** Expression of GFP-YbgC fusions driven by P*tac*. Expression levels of GFP fused to indicated YbgC were visualized and photographed with a confocal microscope. Signal intensities were quantified as described in Methods and presented as relative levels, by normalized to the average of the GFP-SoYbgC sample with 0.05 mM IPTG. *Hi*, *H. influenzae*, *Hp*, *H. pylori*, and *Ec*, *E. coli*. **B.** Western blotting against GFP and GFP fusions from samples prepared in **A.** Cropped blots were shown. Arrows point to target proteins. **C.** Complementation of GFP-*So*YbgC. The experiment was carried out with 0.05 mM IPTG.

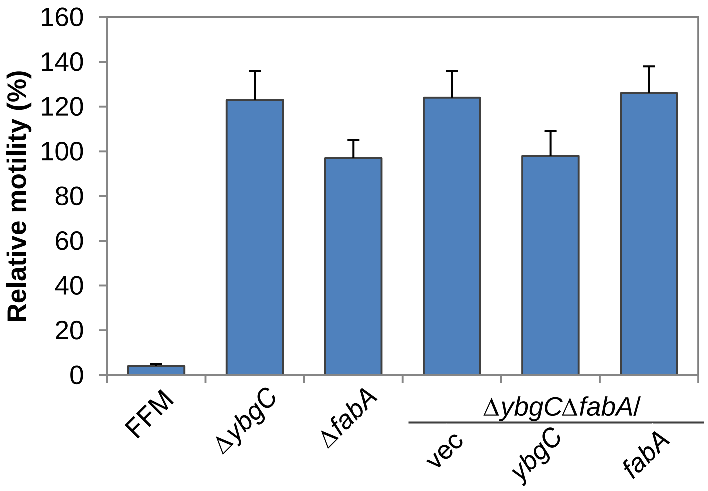

FIG S7. DSF unlikely has a role in *So*YbgC-mediated regulation of motility. Signal molecules of the DSF family studied to date are *cis*-2-unsaturated fatty acids of different chain lengths and branching, synthesized from immediate acyl-ACP substrates of fatty acid biosynthesis, a process relying on FabA. The *fabA* gene under P*tac* was expressed in the Δ*ybgC* and Δ*ybgC*Δ*fabA* strains in the presence of 0.05 mM IPTG. Clearly, the Δ*SoybgC* strain devoid of *fabA* had a similar hypermotile phenotype. The experiment was performed at least three times with the standard error of the mean presented as error bars.

Additional data:

In the *S. oneidensis* proteome, homologues to enzymes that required for DSF synthesis, such as *Xanthomonas* RpfF and RpfB, are not found, Implying that *S. oneidensis* may not produce DSF molecules similar to *cis*-2-unsaturated fatty acids. Despite this, we determined if *S. oneidensis* produces DSF by testing effects of the cell-free spent medium recovered from mid-log and stationary phase cultures of Δ*ybgC* overexperesssing the *ybgC* gene on motility. Cells of the wild-type and Δ*SoybgC* strains at the mid-log phase were collected and inoculated into spent media and motility in terms of swimming speed was monitored in a time-course manner. However, during the treatment up to 4 h the average speed of 100 cells for the wild-type and Δ*SoybgC* strains was not significantly altered (51 $\pm$ 15 and 66 $\pm$ 18 μm/s, respectively), suggesting a lack of DSF in spent medium under test. Thus, *S. oneidensis* is unlikely to produce DSF molecules.

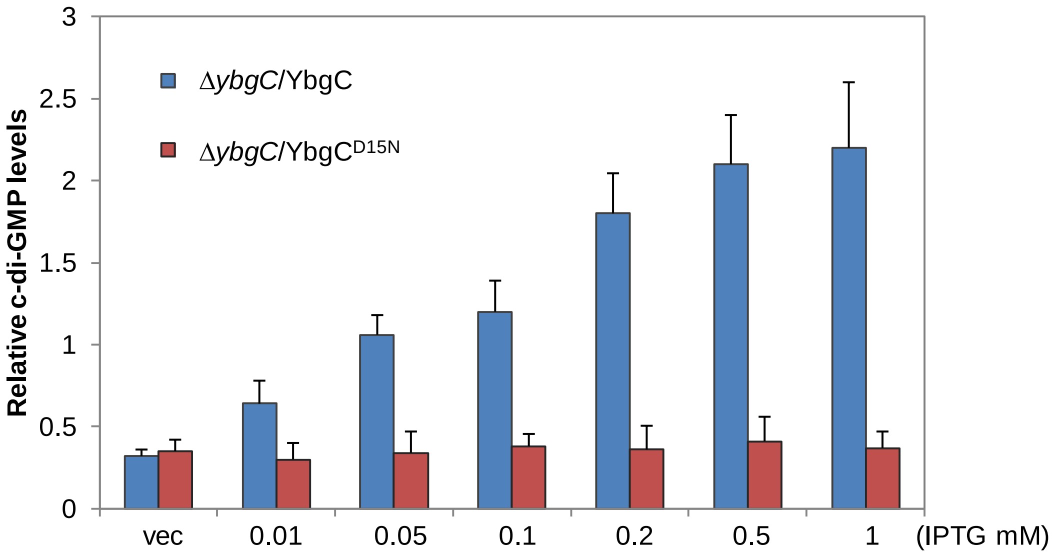

FIG S8. *So*YbgC mediates c-di-GMP levels. Relative intracellular levels of c-di-GMP measured by LC-MS/MS. The Δ*ybgC* strain produced YbgC and $YbgC^{D15N}$ under P*tac* in the presence of IPTG at indicated levels. The levels of c-di-GMP in WT carrying the empty vector (vec) were set to 1 for normalization. Error bars represent the standard error of the mean for at least three biological replicates.
